# Supplementary material for: Hierarchical-Concatenate Fusion TDNN for sound event classification
Source: PLoS One. 2024 Oct 31;19(10):e0312998. doi: 10.1371/journal.pone.0312998 (PMC11527289; doi:10.1371/journal.pone.0312998)
Supplement: Attachment — Submitted filename: Response to Reviewers.pdf [file pone.0312998.s001.pdf]

Manuscript ID: PONE-D-23-39866

Title: Hierarchical-Concatenate Fusion TDNN for Sound Event Classification

Author name: Baishan Zhao, Jiwen Liang

Dear Ali Haider Khan ,

We thank the editor and reviewers for their careful reading of our paper. We have now revised the manuscript and our main revisions include:

(a) supplementing the description of challenges faced by researchers, research objectives, and research contributions in introduction section;

(b) redesigning the HCM network architecture in HCM network subsection;

(c) supplementing the description of operation of HCM Network in split and concatenate operation of HCM network subsection;

(d) adding descriptions of the Sensitivity indicator and Kappa indicator in the experimental setup section of the main text and calculating the Sensitivity indicator and Kappa indicator to assess the prediction performance of the HCM-TDNN on the Urbansound8K dataset in tables 5;

(e) adding ablation experiments on the Urbansound8K dataset in the sound event classification model section;We also provided an itemized response attached to this submission;

(f) supplementing the description of model performance analysis in Comparative Analysis.

We also provided an itemized response attached to this submission.

We have carefully addressed all the concerns raised by the reviewers and believe that our revised manuscript substantially improves upon the initial submission. We hope that the reviewers and editor find our revisions satisfactory and consider our manuscript for publication in ***Plos one***.

Warm regards,

Jiwen Liang, Ph.D.
School of Information Science and Engineering, Shenyang University of Technology
111 Shenliao West Road, Economic and Technological Development, Shenyang, Liaoning Province, China
Telephone Number: +8618624341326
E-mail Addresses: a17741226289@163.com

Reply to the comments

All the revisions have been marked in blue for a point-by-point response to the editor and reviewers' comments and highlighted in the manuscript with yellow background. All page numbers refer to the revised manuscript and below are our responses to the specific comments raised:

Comments from editor:

1. Comment: The research problem article is addressing needs to be clearer and must be defined in the introduction section clearly.

Author Response:

Thank you for your feedback regarding the clarity of the research problem addressed in our manuscript. We acknowledge the importance of presenting a clear and well-defined research problem in the introduction section.

We have re-written this part according to the editor. Specific modifications are as follows:

we have corrected the "*To solve the above problem, HCM is proposed* " into"*Aiming at the limitations of the ECAPA-TDNN in capturing extensive receptive fields and the problems causes by existing models in wide-range or multi-scale sound events, this paper proposes a Hierarchical-Concatenate Model (HCM) integrating the TDNN network.*" (please see: p.2, Line 33-36).

2. Comment: The research objectives are not defined clearly in the introduction section.

Author Response:

Thank you for your meticulous review and invaluable feedback on our manuscript.

We acknowledge the concern you raised regarding the clarity of the research objectives within the introduction section. We have revised the following text in our introduction.

(a)In the introduction section, we have supplemented a discussion about the challenges faced by existing research methods. This provides background and justification for the direction and objectives of this paper(please see: p.2, Lines 23-32).

(b)In the introduction section, we have clearly stated our specific aims and objectives: *Aiming at the limitations of the ECAPA-TDNN in capturing extensive receptive fields and the problems causes by existing models in wide-range or multi-scale sound events, this paper*

proposes a Hierarchical-Concatenate Model (HCM) integrating the TDNN network.(please see: p.2 lines 33-36).

(c)In the introduction section, the contributions made by this study have been added. This addition aims to help readers better understand the core objectives of the article(please see: p.2 lines 42-49).

"*Key contributions of this study include: (1) Novel Network Design: Proposing HCM, focusing on the effective fusion of multi-scale information to better capture both local and global features within Mel spectrograms. (2) Multi-scale Information Fusion: Through parallel convolutional layers in HCM, the model adeptly manages features of varying scales, significantly boosting the recognition of micro & macro features within Mel spectrograms. (3) Experimental Validation: This study contrasts HCM-TDNN with three prominent existing convolutional neural network models on the standard Urbansound8K dataset, demonstrating its state-of-the-art performance.*"

3. Comment: There is a serious concern, authors should conduct a comparative analysis to prove that their research has better performance, and addressing all the objectives of this proposed system.

Author Response:

we express our heartfelt gratitude for your detailed feedback. Regarding your significant concerns about conducting a comparative analysis to prove the superiority of our research in performance and consistent with all the objectives of the proposed system, we have thoroughly considered and formulated the following response:

(a) Performance Comparison Analysis: The sensitivity metric and the Kappa metric have been added to further evaluate the proposed system(please see: p.11, Table 5).

(b) Validation of System Objectives: (1)We have supplemented a boxplot of the F1 score to provide readers with a more intuitive comprehensive evaluation of the model's performance(please see: p.10, Fig 5); (2)We have included a boxplot of the Kappa, highlighting that our model excels in consistency and predictive accuracy(please see: p.10, Fig 6). (3)We have increased the number of training epochs from 30 to 50 and have supplemented loss curves to visually assess whether the HCM model is overfitting or underfitting(please see: p.10, Fig 7); (4)We have redrawn the accuracy to further analyze the model's classification accuracy (please

see: p.11, Fig 8);

(c) Experimental Design: (1)We conducted activation function comparison experiments. The improvement in system accuracy with ReLU, Leaky ReLU, and PReLU has been focused on in the analysis(see p.7, Table 3); (2)We have added ablation experiments for the HCM-TDNN network framework to evaluate the contribution of each component in the model(see p.8, Table 4).

(d) Results Interpretation: We have included additional experimental results and discussion to provide a more detailed consideration for the HCM-TDNN system(please see p.8, Line 211-230).

"*To further evaluate the performance of the four different models in sound event classification, a box plot is generated to visualize the distribution of F1 scores for each model, as shown in Fig 5.*

Through the analysis of the box plot results for F1 scores, we can clearly compare and interpret the performance of various models in audio classification. It can be seen from median F1 scores that the HCM-TDNN model and ECAPA-TDNN exhibit similar performance. Therefore, it is necessary to further assess the models comprehensively using Kappa values, providing a more holistic evaluation than simple accuracy, especially in cases of class imbalance or uneven errors, as shown in Fig 6.

It should be noted that, for the median Kappa values, the HCM-TDNN model demonstrates the highest predictive consistency. Additionally, the HCM-TDNN model exhibits relatively short whiskers, indicating stable performance."

Table3. The model accuracy and F1 score under different activation functions.

Activation Function	Networks	Accuracy	F1-Scores
Relu	HCM-TDNN	94.44%	95.92
Leaky ReLU	HCM-TDNN	94.79%	95.88
PReLU	HCM-TDNN	95.83%	97.04

This table shows the accuracy results and F1 scores of each activation function on the HCM-TDNN.

Table4. Ablation study of the HCM-TDNN architecture.

Experimental	SE Block	HCM Block	Skip Connections	Accuracy	Precision	Sensitivity	Recall	F1-scores
Exp.1	×	×	×	84.72%	85.14%	98.36%	83.75%	84.44
Exp.2	×	×	√	85.42%	85.77%	98.43%	87.68%	86.71
Exp.3	×	√	×	88.54%	87.73%	98.8%	92.5%	90.58
Exp.4	√	×	×	87.85%	89.28%	98.7%	90.39%	89.83
Exp.5	×	√	√	89.24%	90.48%	98.8%	92.5%	91.48
Exp.6	√	×	√	89.58%	89.98%	98.81%	91.96%	91.67
Exp.7	√	√	×	93.40%	94.16 %	99.34 %	96.04 %	95.09
Exp.8	√	√	√	95.83%	96.59 %	99.6%	97.5%	97.04

This table shows the results of the HCM-TDNN network ablation experiment.

Table5. Comparison of Papers

Datasets	Method	Accuracy	Precision	Sensitivity	Recall	F1-scores	Kappa
UrbanSound8K	Conformer	81.25%	84.23 %	83.21%	79.14 %	81.61	0.7943
	ECAPA-TDNN	90.625%	91.33 %	92.44%	93.75 %	92.52	0.9103
	AF-TDNN	92.014%	93.09 %	94.87%	94.21 %	93.65	0.923
	HCM-TDNN	95.83%	96.59 %	99.6%	97.5%	97.04	0.9615

This table indicate that the testing performance of the HCM-TDNN model is superior to other network models.

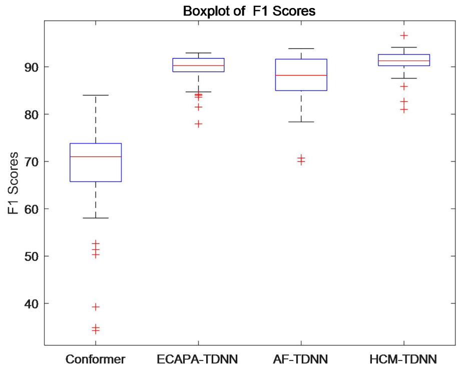

Fig 5. Comparison Box Plot of F1 Scores

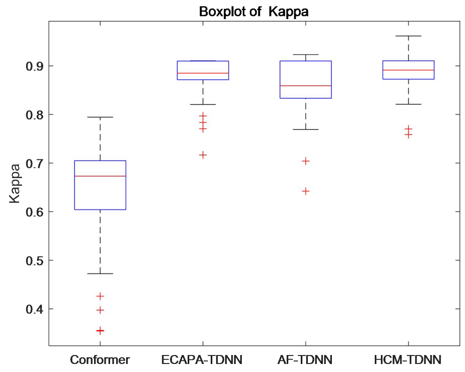

Fig 6. Comparison Box Plot of Kappa

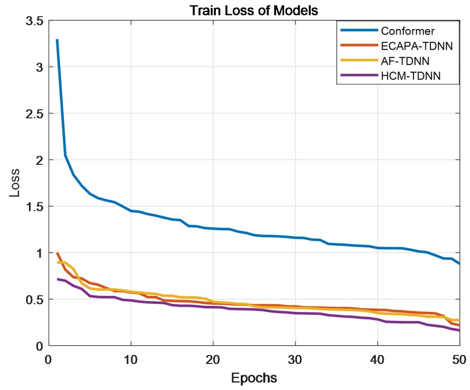

Fig 7. Loss curves of each model

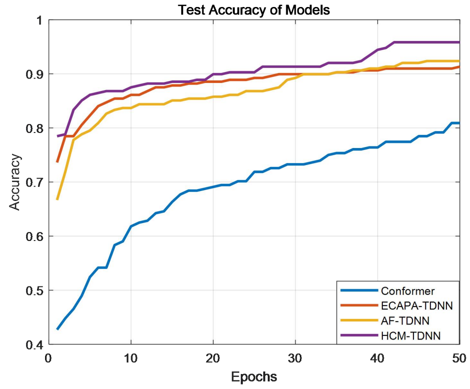

Fig 8. Model testing accuracy curves based on UrbanSound8K Datasets

4. Comment: Methodology relating to the objectives of this research is not satisfactory, need revision to fulfill all the objectives of this research.

Author Response:

Thank you for highlighting your concerns regarding the methodology. As you are concerned, there are several problems that need to be addressed. According to your nice suggestions, we have made extensive corrections to our previous draft, the detailed corrections are listed below.

(a) We have redesigned the HCM network architecture to help readers better understand the operational processes within the HCM network structure(please see: p.5, Fig 3).

(b) We have revised the formulas in the methodology section(please see: pp.4-5, Line 89-111).

"*The feature maps are divided into* s *groups, represented by* x_1, x_2,..., x_s. *Each grouphas consistent channel width, denoted by* ω. *Subsequently, each* x_1 *is processed via* s *convolutional operations. These convolutions can be represented as* G_1, G_2,..., G_s. *When we perform convolutional operations on* x_1, x_2,..., x_s, *the output feature maps for each* x_1, x_2,..., x_s *are represented as* y_1, y_2,..., y_s. *The innovation is an introduction to a novel concept, instead of processing these output feature maps independently, we introduce anovel concept. We concatenate adjacent feature maps* y_i *and* y_{i+1} *to form a sub-group, represented as* $y_{i,i+1}$. *Mathematically, for each* y_i *and* y_{i+1}, *the concatenation operation is formulated as:*

$$y_i = G_i\left(x_i\right) \qquad i = 1, 2, ..., s \tag{1}$$

$$y_{i,i+1} = Concat\left(y_{i-1}, y_i\right) \qquad i = 2, ..., s \tag{2}$$

Where, " Concat " denotes the concatenation of two feature maps along the channel dimensions. After pairwise concatenation, $s-1$ *sub-groups are formed. And then, these sub-groups are fed into a subsequent series of layers for further convolution and adjacent feature map concatenation until forming a single sub-group. Similarly, in Fig 3,* $z_{i,i+1}$, $o_{1,2,...,i,i+1}$, *and* $o_{i,i+1,...,s-1,s}$ *represent the results of subgroup convolution operations. After operating all input feature groups,* $o_{1,2,...i,...s-1,s}$ *restores the channel dimension by concatenating all and then utilizing 1D convolution to reduce the dimensionality. The result after dimensionality reduction is denoted by* O.

The method of creating sub-groups by concatenating adjacent feature maps within each group enhances the model's ability to capture both local and contextual information. It provides a more structured features, which can improve performance in sound classification by convolutional neural networks."

(c) We have added subsections to the methodology to describe the split and concatenate

operations.(please see: p.5, Line117-137).

The Hierarchical Concatenation Module (HCM) consists of two primary stages: the split and concatenate operations.

• Splitting Operation: Initially, the integrated feature map X is divided into different groups, denoted by x_i Importantly, each of these groups xi maintains the same width.

• Phase of Connection Operation: This operation combines adjacent feature maps y_i and y_{i+1} to form a sub-group $y_{i,i+1}$. It's worth noting that the channel width of $y_{i,i+1}$ is twice y_i or y_{i+1} .

• Purpose of Identity Mapping: The identity mapping for y_i aims to preserve the intrinsic feature maps. Conversely, $y_{i,i+1}$ focus on capturing more complex features.

We are committed to enhancing the reproducibility of the methodological aspects to fulfill the research objectives in our manuscript. Based on your comments, we have made the corrections to make the unit fulfill all the objectives of this research.

Once again, we appreciate your constructive feedback.

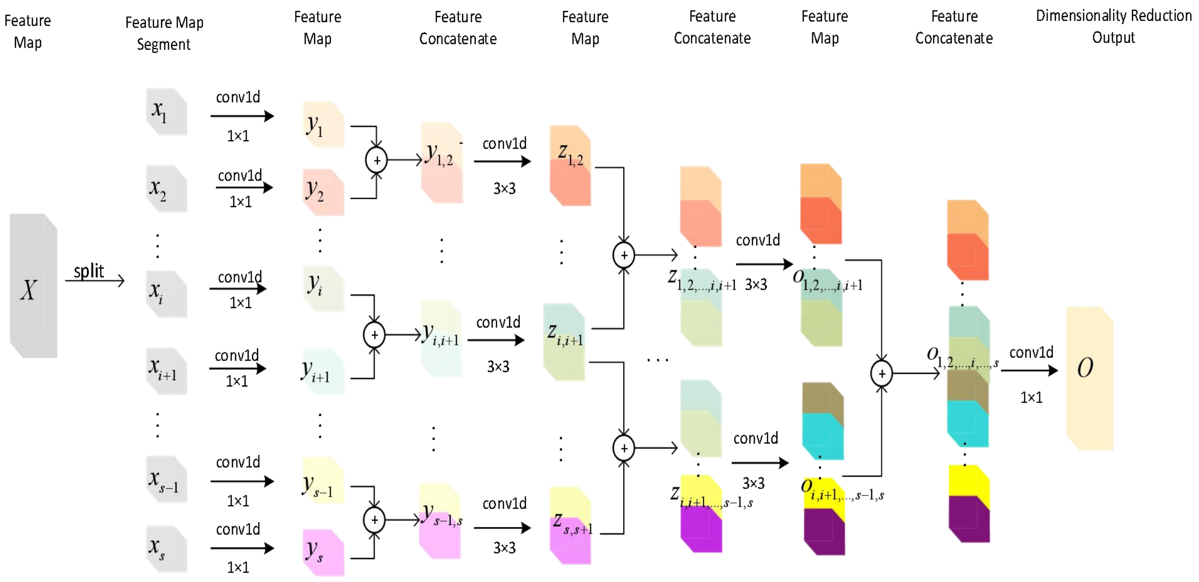

Fig 3. A Detailed View of the HCM

Comments from Reviewer:

Reviewer #1:

1. Comment: The author claims that "to solve the above problem, HCM is proposed," but the author did not specify the above problem.

Author Response:

We Thank the reviewer for pointing this out.

We have corrected the "*To solve the above problem, HCM is proposed* " into "*Aiming at the limitations of the ECAPA-TDNN in capturing extensive receptive fields and the problems causes by existing models in wide-range or multi-scale sound events, this paper proposes a Hierarchical-Concatenate Model (HCM) integrating the TDNN network.*" (please see: p.2, Line 33-36).

Your feedback is valuable. We have ensured that this clarification is adequately addressed in the revised version of the paper.

2. Comment: The authors did not follow English rules. There should be some space between two sentences, e.g., (I went to shop. I bought eggs.).

Author Response ：

Thank you for pointing out this issue. Regarding the spacing between sentences, we acknowledge the oversight.

The English rules has been improved. The appropriate spacing between sentences throughout the entire manuscript has been accordingly adjusted.

Your feedback is appreciated. We have made the necessary adjustments to enhance the readability and compliance of the manuscript.

3. Comment： All the text should be aligned, which does not seem to be the case in the research.

Author Response:

Thank you for bringing this to our attention.

In the revised version, we have ensured that all text is properly aligned throughout the document.

Your feedback is valuable, and we have made the necessary adjustments to address this issue effectively.

4. Comment: The authors did not mention their contributions specifically in the current research.

Author Response:

Thank you for your valuable feedback regarding the clarity of the authors' contributions in our manuscript. In response to these concerns, we have revised the manuscript. Specifically:

"*Key contributions of this study include(please see: p.2, lines 42-49): (a)Novel Network Design: Introducing HCM, focusing on the effective amalgamation of multi-scale information to better capture both local and global features within Mel spectrograms. (b)Multi-scale Information Fusion: Through parallel convolutional layers in HCM, the model adeptly manages features of varying scales, significantly boosting the recognition of micro and macro features within Mel spectrograms. (c)Experimental Validation: This study contrasts HCM-TDNN with three prominent existing convolutional neural network models on the standard Urbansound8K dataset. The recognition accuracy of the HCM-TDNN model reached 95.83%. This marks an improvement of approximately 5% compared to the baseline model's performance.*"

By providing this clarification, we aim to offer a clear understanding of the research in this article.

We appreciate your constructive feedback, which has helped enhance the quality and clarity of our manuscript.

5. Comment: Overall, this research sounds good.Recommended with minor changes.

Author Response:

Thank you for your positive feedback on our manuscript titled "Hierarchical-Concatenate Fusion TDNN for Sound Event Classification."

We are pleased to learn that you found the research overall sound. We have implemented the suggested revisions to enhance the manuscript. The attached document includes the updated version, with changes clearly highlighted for your convenience.

Reviewer #2:

1. Comment: In figure 4, except conformer, all the methods showing and achieved same

accuracy till epoch 29, justify it?

Author Response:

Thank you for pointing out the observation regarding Fig 4 in our manuscript. You've highlighted that, until epoch 29, all methods, excluding the conformer, displayed and achieved the same accuracy. Allow me to provide some justifications and insights into this phenomenon:

(a) Initial Model Training and Learning Rate Adjustments: During the early stages of model training, it's plausible for various methods to converge to similar performance levels. This convergence can be attributed to weight initialization and specific learning rate settings.

(b) Characteristics of the Dataset: Certain inherent complexities or patterns within the dataset might cause multiple methods to exhibit comparable performances at a specific phase. These complexities or patterns can influence the convergence of various methods during that particular phase. This could be attributed to certain shared patterns or features that are effectively captured across these methods.

(c) Further Experiments and Analysis: To further explore and explain this phenomenon, we have extended the experiments and analysis from epoch=30 to epoch=50(please see: p.10, Fig 8). Additionally, We have introduced kappa metrics, sensitivity, and loss curves to further evaluate the reliability of the model.

In summary, we believe that this phenomenon could be a confluence of multiple factors and intricacies inherent to the dataset. We are committed to conducting further investigations to offer a more detailed and nuanced explanation.

Once again, we sincerely appreciate your valuable feedback and insights, which significantly contribute to refining our research.

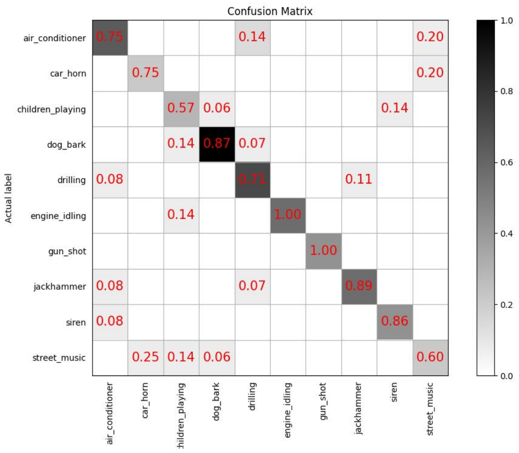

(a)

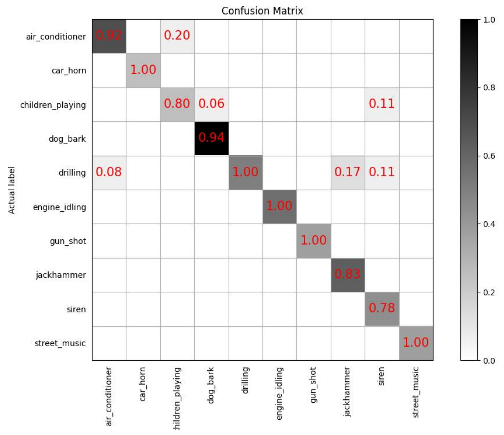

(b)

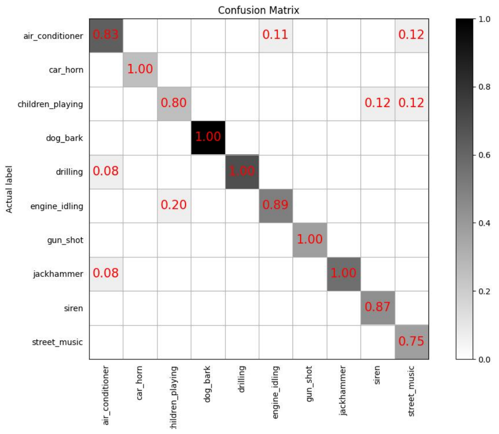

(c)

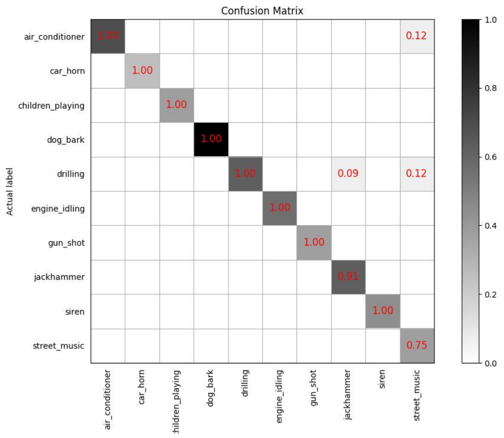

(d)

Fig 4. Confusion matrix for (a) Conformer, (b) AF-TDNN, (c)ECAPA-TDNN and (d)HCM-TDNN.

2. Comment: is the code for this experiment is publicly available for authenticity?

Author Response:

Thank you for your inquiry regarding the availability of our experimental code for ensuring authenticity.

The code had been developed for a certain technology company. Unfortunately, we cannot currently make the code associated with this experiment publicly available. This restriction is due to the need for company intellectual property.

Should you have any further questions or require additional information about the code or our methodologies, please do not hesitate to let us know.

Thank you once again for your valuable feedback and for emphasizing the importance

of authenticity in scientific endeavors.

3. Comment: why you use the technique Mel spectrograms as input features in this proposed network?

Author Response:

Thank you for your insightful question regarding our choice of using Mel spectrograms as input features in the proposed network.

The decision to employ Mel spectrograms as input features stems from several reasons:

(a) Frequency Representation: Mel spectrograms offer a logarithmic frequency representation that aligns more closely with human auditory perception. This representation is particularly beneficial for tasks related to sound or speech processing. It can capture essential frequency components effectively.

(b) Feature Discrimination: Mel spectrograms encapsulate critical spectral details in a compact format. By leveraging this representation, our network can potentially extract more discriminative features. This could enhance its performance in tasks such as sound event detection or classification.

(c) Empirical Performance: Preliminary experiments and prior research in the field have indicated that Mel spectrograms can yield favorable results. These results were observed when Mel spectrograms were used as input features for similar neural network architectures. This suggests the potential effectiveness of Mel spectrograms in enhancing neural network performance. By adopting this approach, we aim to build upon existing knowledge and leverage proven techniques to achieve robust performance.

(d) Computational Efficiency: Mel spectrograms provide a computationally efficient representation of audio signals. This efficiency can be crucial, especially when dealing with large datasets or real-time processing requirements.

In summary, our choice of using Mel spectrograms as input features is rooted in their alignment with auditory perception, their ability to capture critical spectral details, empirical evidence from prior studies, and considerations of computational efficiency.

We deeply appreciate your meticulous attention to detail, which have undoubtedly strengthened the overall quality of our manuscript.

4. Comment: Have you check the accuracy of your proposed techniques on lesser or more epoch(i.e at 20-25 or at 35-40) ?

Author Response:

Thank you for your insightful question regarding the evaluation of our proposed techniques across different epochs.

Yes, we have indeed experimented with varying the number of epochs during our validation process to assess the robustness and performance stability of our proposed techniques. Specifically:

Fewer Epochs (20-25): We conducted experiments by limiting the training epochs to a range of 20-25. However, unfortunately, we did not achieve the accuracy levels mentioned in other researchers' papers.

More Epochs (35-40): Similarly, we extended our training sessions to encompass epochs between 35-40. The results of these experiments revealed that increasing the number of epochs did not further enhance the model's test accuracy. On the contrary, as the epochs increased, so did the training duration. To validate our description, in the revised manuscript, we extended the epochs from 30 to 50 to intuitively observe the changes in the model's test accuracy(please see: p.10, Fig 8).

We sincerely value your expertise, which has played a crucial role in shaping the final version of our manuscript. Your thorough review has been indispensable in elevating the quality of our research.

Overall Changes Made:

Based on the reviewers' feedback, we have made the following major revisions:

1. INTRODUCTION

(a) We have added an evaluation of existing research work, acknowledging the contributions made by researchers and the challenges they have faced.

(b) We have corrected the "To solve the above problem, HCM is proposed " into "Addressing the limitations of the ECAPA-TDNN in capturing extensive receptive fields and the challenges posed by existing models in handling broad or multi-scale sound events"

(c) Specific contributions of this study have been added to the introduction.

(d) Highlight all the modifications made.

2. SOUND EVENT CLASSIFICATION MODEL

(a) We have redesigned the framework diagram of the HCM-TDNN network and the HCM network framework diagram.

(b) We have redesigned the structure diagram of the HCM network.

(c) We have redefined the calculation formula and framework details of the HCM network structure

(d) We have added new subsections to specifically describe the splitting and cascading operations of the HCM network.

(e) Highlight all the modifications made.

3.EXPERIMENTAL SETUP

(a) We have increased the epoch from 30 to 50. With the gradual increase of epochs, the model has learned more features and patterns. On the test data, the accuracy of the model has reached 95.83%.

(b) Highlight the modified parts

4. EXPERIMENTAL RESULTS

(a) We have added the Kappa and sensitivity metrics to the original evaluation criteria.

(b) Under the condition of Epoch=50, we have added the plotting of the loss curve and have redrawn the accuracy curve.

(c) We have plotted boxplots for F1 score and Kappa.

(d) In addition to the confusion matrix of the HCM model, we have added confusion matrices for the other three models in the article.

(e) We have added ablation experiments

(f) All modified sections are highlighted.

5.Others

(a) Polish the content of the article: The proofreading of the article has involved correcting grammar, adjusting tenses, and rectifying professional terminology.

(b) Article formatting: The formatting of the article has involved modifications, including text alignment and paragraph formatting.

Conclusion:

Thank you once again for the constructive feedback and consideration. We look forward to your positive response.
