# Supplementary material for: Hierarchical-Concatenate Fusion TDNN for sound event classification
Source: PLoS One. 2024 Oct 31;19(10):e0312998. doi: 10.1371/journal.pone.0312998 (PMC11527289; doi:10.1371/journal.pone.0312998)
Supplement: Attachment — Submitted filename: Response to Reviewers.pdf [file pone.0312998.s003.pdf]

Manuscript ID: PONE-D-23-39866R1

Title: Hierarchical-Concatenate Fusion TDNN for Sound Event Classification

Author name: Baishan Zhao Jiwen Liang

Reply to the comments

Dear Ali Haider Khan ,

Thanks the editor and reviewers again for their careful reading of our paper. We have carefully considered these comments once again and have made corresponding revisions to the manuscript. Please find below our responses to the reviewers' comments. All the revisions have been marked in blue for a point-by-point response to the editor and reviewers' comments and highlighted in the manuscript with yellow background. All page numbers refer to the revised manuscript and below are our responses to the specific comments raised:

Comments from Reviewer：

Reviewer #1:

(1) Comment: All the comments have been corrected, and the article is now written in proper shape.Recommended to accept.

Author Response:

Thank you for your positive feedback on our manuscript titled "Hierarchical-Concatenate Fusion TDNN for Sound Event Classification."

Reviewer #2:

(1) Comment: The abstract is poorly written. Most of the content focuses on the outcome of the proposed model. It should include a little bit of problem background and research objectives as well.

Author Response:

Thank you for your constructive feedback regarding the abstract.

We acknowledge the shortcomings of the abstract. We have re-written this part according to the reviewer. Specific modifications are as follows:

First, we have corrected the "*Semantic feature combination/parsing issue is one of the key problems in sound event classification.*" into "*Semantic feature combination/parsing issue is one of the key*

problems in sound event classification for acoustic scene analysis, environmental sound monitoring, and urban soundscape analysis."

Second, we have corrected the"*Hierarchical-Concatenate Model (HCM) is proposed to improve the performance of ECAPA-TDNN Networks.*" into "*TThis paper proposes the Hierarchical-Concatenate Fusion(HCF)-TDNN model by adding HCF Module to ECAPA-TDNN model for sound event classification*."

Third, we have corrected the"*Second, the segmented features are convolved one by one. Finally, after the convolution is completed, the two adjacent parts are combined before proceeding with the next convolution.*" into "*Then, the segmented features are convolved one by one for improving the small receptive field in perceiving details. Finally, after the convolution is completed, the two adjacent parts are combined before proceeding with the next convolution for enlarging the receptive field in capturing large targets.*"

Finally, we have added the"*The proposed model is trained and validated on the Urbansound8K dataset.*" and " *which is approximate improvement of 5% (relatively) over ECAPA-TDNN model.*" (please see: Abstract).

Once again, we sincerely appreciate your valuable feedback and insights, which significantly contribute to refining our research.

(2) Comment: The Introduction section should provide a brief introduction for the rest of the article's contents instead of the literature review-related contents.

Author Response:

Thank you for your valuable feedback regarding the Introduction section.

Yes, We acknowledge the need to refocus the Introduction to provide a brief overview for the rest of the article's contents. In the introduction section, the rest of the article's contents have been added. Specifically:

"*The rest of the paper is organized as follows: In section 2, the sound event classification model was described, including the topology of HCM-TDNN and the details and operational principles of the HCM module structure. Section 3 details about dataset and the experimental setup. Section 4 shows results and makes analysis in different challenge part as well. Finally, our works are concluded in section 5*".(please see:p.2 lines 44-48).

Thank you once again for your valuable feedback and for emphasizing the importance of authenticity in scientific endeavors.

(3) Comment: The literature review should be presented comparatively to show the research gap quantitatively (if possible). For instance, the author can identify some parameters and compare

the related models/methodologies based on them in a tabular form.

Author Response:

Thank you for your suggestion regarding the presentation of the literature review.

We acknowledge the importance of presenting the literature review in a comparative manner to highlight quantitative research gaps. We have enhanced our manuscript by comparing related models, as suggested. Specifically:

We have corrected the "*Notably, the ResNet combined with the SE module [11], Convolutional Neural Networks (CNN) [12][13][14], and approaches grounded in Transformer Networks have been explored.* " into " *Notably, the ResNet[13] combined with the SE module [14] to enhance the robustness of classification model. Convolutional Neural Networks (CNN) [15-17] have become the standard method for addressing various audio classification problems. And approaches grounded in Transformer Networks have achieved great success in the text recognition task.*"(please see:p.2 lines 13-18).

Besides, we have revised the " *Furthermore, prevalent neural network architectures are based on Time Delay Neural Networks (TDNN) [16], also known as x-vector architectures.*" into "*Furthermore, prevalent neural network architectures are based on Time Delay Neural Networks (TDNN) [19], which widely used in Automatic Speaker Verification, such as x-vector system.*" (please see:p.2 lines 19-21).

(4) Comment: The research objectives, aims, motivations, and ultimate beneficiaries should be well explained to show the significance of the research contribution.

Author Response:

Thank you for your valuable feedback regarding the clarity of the research objectives, aims, motivations, and ultimate beneficiaries.

We have carefully read all suggestions by the reviewer and added corresponding descriptions of the research objectives, aims, motivations, and ultimate beneficiaries in the introduction section on page 2.

We have revised the"*Key contributions of this study include: (1) Novel Network Design: Proposing HCM, focusing on the effective fusion of multi-scale information to better capture both local and global features within Mel spectrograms. (2) Multi-scale Information Fusion: Through parallel convolutional layers in HCM, the model adeptly manages features of varying scales, significantly boosting the recognition of micro & macro features within Mel spectrograms*."into"*Meanwhile, key contributions of this study include: (1) Novel Model Design: Introducing HCF module, focusing on the effective amalgamation of multi-scale information to better capture both local and global features within Mel spectrograms to help this model get higher evaluation metrics. (2) Multi-scale Information Fusion: Parallel convolutional layers are used for multi-scale feature information fusion in HCF module to make the detection system more robust in different tasks*".(please see:p.2 lines 36-41).

We sincerely value your expertise, which has played a crucial role in shaping the final version of our manuscript. Your thorough review has been indispensable in elevating the quality of our research.

(5) Comment: The graphics quality of figures should be improved so that the textual information should be better readable.

Author Response:

Thank you for your feedback regarding the graphics quality of the figures.

We acknowledge the importance of enhancing the clarity and readability of the textual information within the figures. We have improved the quality of full-text images by increasing the image resolution, using vector graphics, redrawing images and other methods.

In this updated version, we improved figures 1-8 in the main text. Details can be found in (Fig 1, page 3; Fig 2, page 3; Fig3, page4; Fig4, page9; Fig5, page10; Fig6, page10; Fig7, page11; Fig 8, page 11).

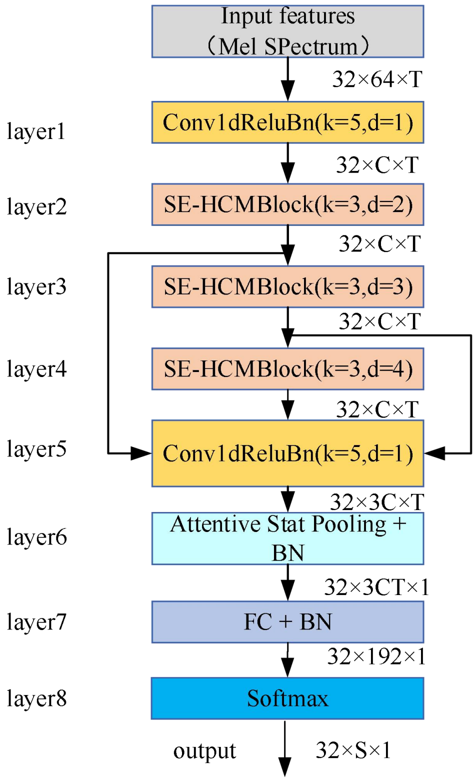

Fig 1. A network architecture with the sound event classification model proposed by HCM-TDNN.

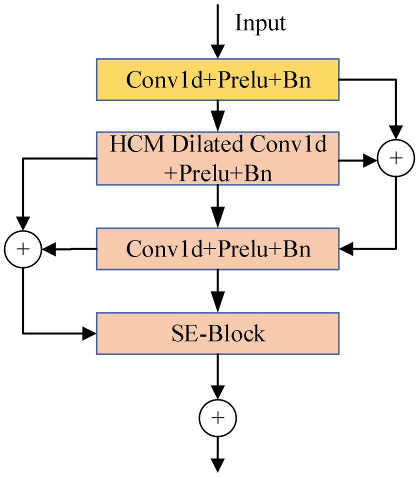

Fig 2. The SE-HCM Block of the ECAPA-TDNN architecture.

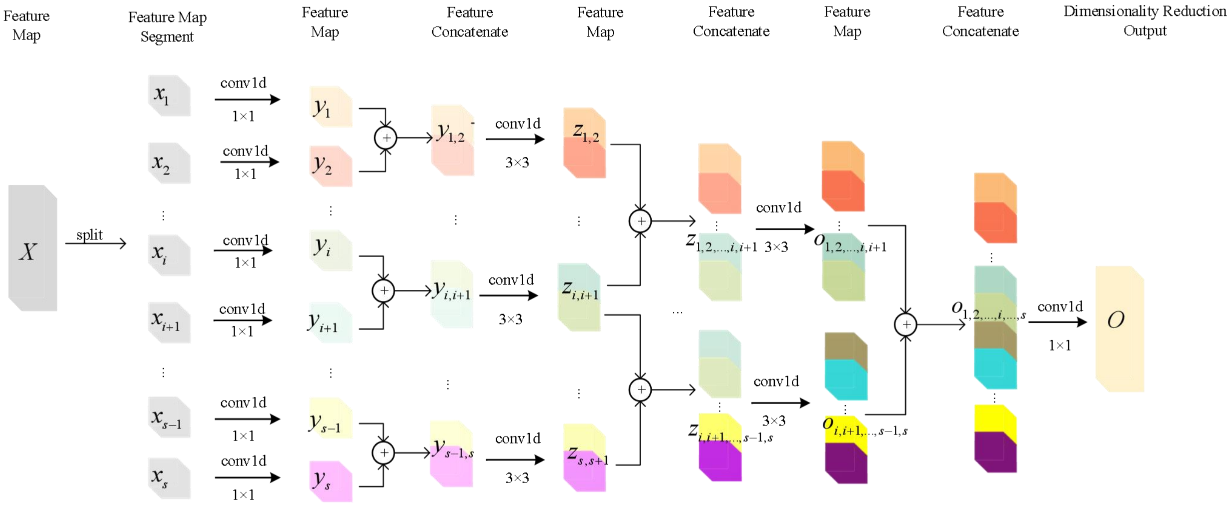

Fig 3. A Detailed View of the HCM.

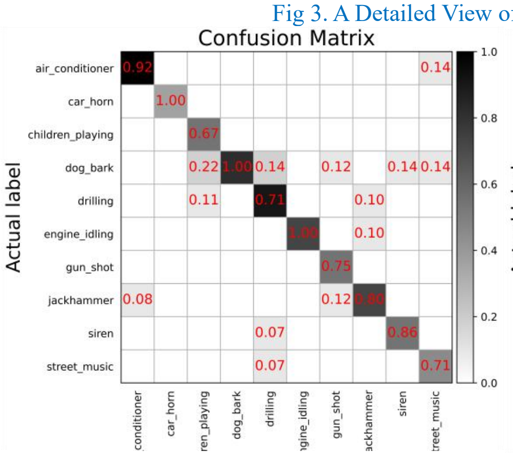

(a) Conformer

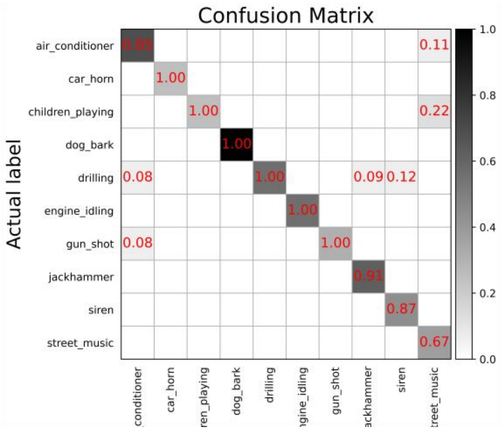

(b) ECAPA-TDNN

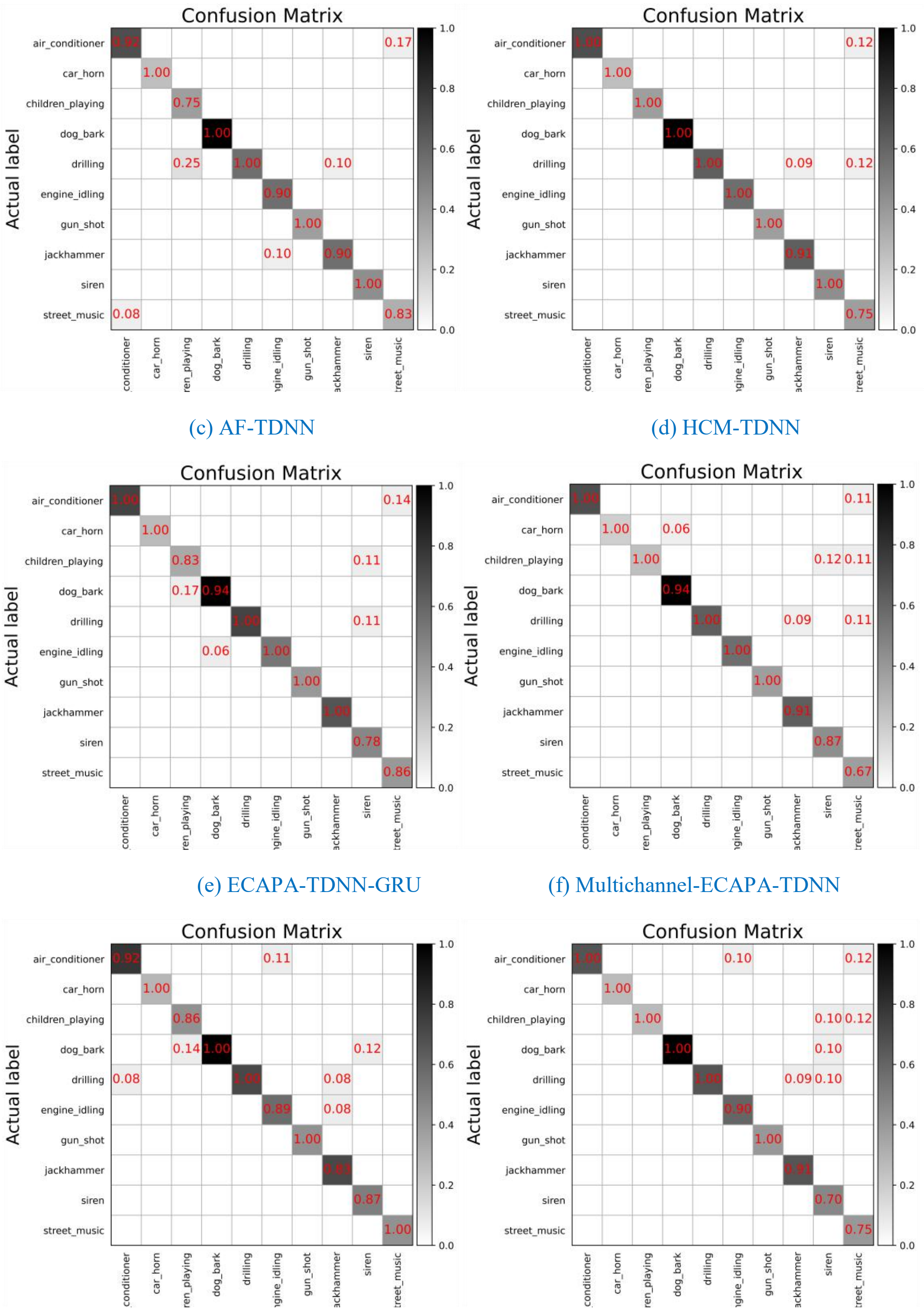

(c) AF-TDNN

(d) HCM-TDNN

(e) ECAPA-TDNN-GRU

(f) Multichannel-ECAPA-TDNN

(g) VGG19

(h) STF-YoLo8

Fig 4. Comparison of confusion matrices for eight models in sound classification.

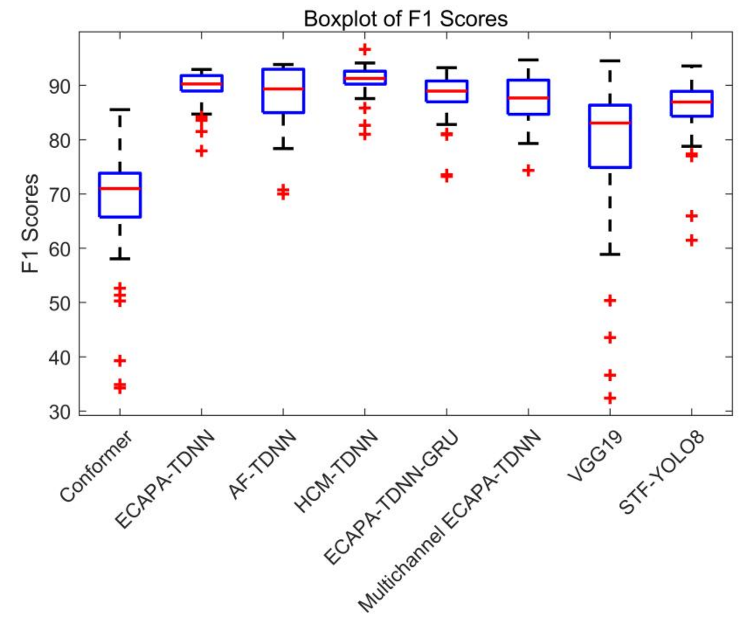

Fig 5. Box plot analysis of F1 scores for various models.

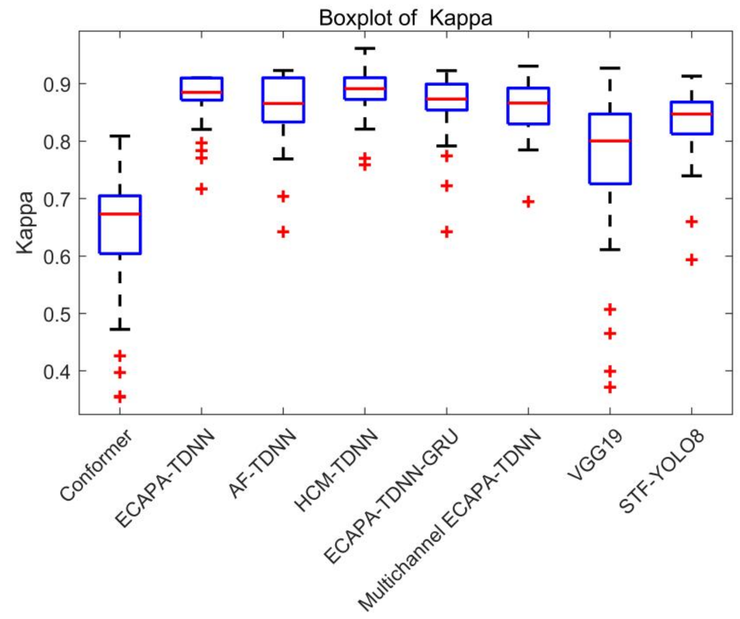

Fig 6. Comparison box plot of Kappa values for different models in sound classification.

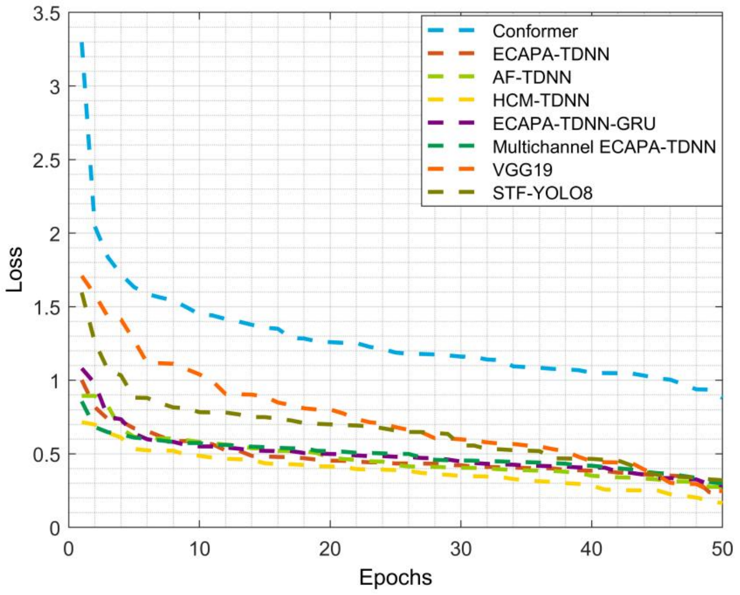

Fig 7. Comparison of Loss curves across various models on UrbanSound8K dataset.

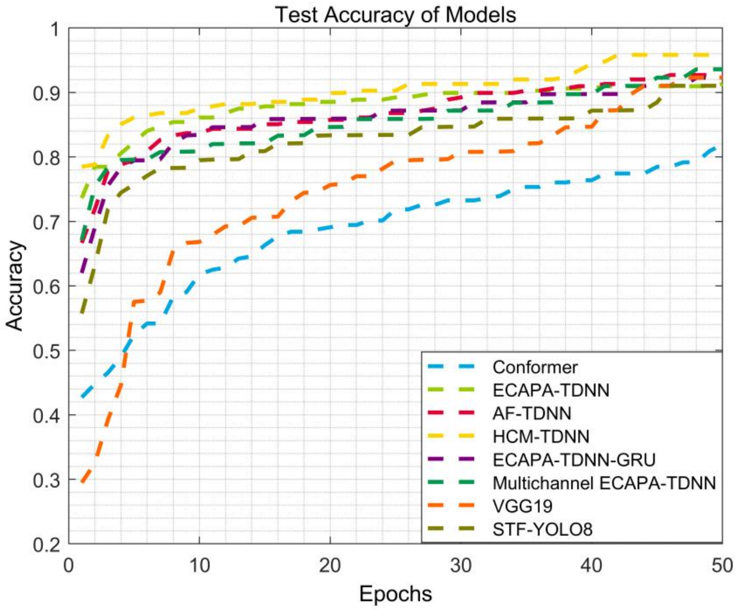

Fig 8. Model testing accuracy curves based on UrbanSound8K dataset.

(6) Comment: It is suggested to add a little bit more description after each figure instead of giving their labels only. For reference, please see figures from numbers 4 to 7.

Author Response:

Thank you for your valuable suggestion. We have revised the manuscript to include more detailed descriptions after figures 4 to 7. Specifically：

"We have changed the captions of Fig 4, Fig 5, Fig 6 and Fig 7 from " *Confusion matrix for (a) Conformer, (b) AF-TDNN, (c)ECAPA-TDNN and(d)HCM-TDNN.*" to " *Comparison of Confusion Matrices for Eight Models in Sound Classification.*" (please see: p.9), " *Comparison Box Plot of F1 Scores.*" to " *Box Plot Analysis of F1 Scores for Various Models.*" (please see:p.10), " *Comparison Box Plot of Kappa.*" to " *Comparison Box Plot of Kappa Values for Different Models in Sound Classification.*" (please see:p.10) and " *Loss curves of models.*" to " *Comparison of Loss Curves Across Various Models on UrbanSound8K Dataset.*" (please see:p.11).

(7) Comment: The two headings are being repeated. See line number 50 and 181.

Author Response:

Thank you for pointing out the repetition of the two headings. We have revised the document to ensure that each heading is unique and that there are no repetitions throughout the text. Specifically：

The heading "*SOUND EVENT CLASSIFICATION MODEL*" has remained unchanged. (please see:p.2 lines 49). And the heading "*SOUND EVENT CLASSIFICATION MODEL*" has been changed to "*EXPERIMENTAL RESULTS*". (please see:p.7 lines 181).

(8) Comment: Future directions should also be discussed to explain the possible future advancements in the underlined research area.

Author Response:

Thank you for your suggestion. We agree that discussing future directions is important to explain the possible future advancements in the research area.

We have supplemented "In future work, the voice recognition system HCM-TDNN model will be deployed in edge computing devices for real-time urban environmental sound recognition scenarios." (please see:p.12 lines 248-250).

(9) Comment: The referenced articles are fewer in number to better support the importance and significance of the underlying study. It is recommended to increase the number of referenced articles by adding more related models/methodologies to be compared with the proposed one.

Author Response:

Thank you for your valuable feedback. We recognize the need to increase the number of referenced articles to better support the importance and significance of our study.

In the revised manuscript, We have added more relevant models and expanded the list of cited articles. The number of citations has increased by 8 compared to the first revised manuscript, bringing the overall number to 32. Particularly:

[1] Mashhadi MMR, Osei-Bonsu K. Speech Emotion Recognition Using Machine Learning Techniques: Feature Extraction and Comparison of Convolutional Neural Network and Random Forest. PLOS ONE. 2023 Nov;18(11):e0291500.

[2] Yue Z, Loweimi E, Christensen H, Barker J, Cvetkovic Z. Acoustic Modelling from Raw Source and Filter Components for Dysarthric Speech Recognition. IEEE/ACM Trans AudioSpeech and Lang Proc. 2022 Sep;30(1):2968-2980.

[3] Du XW, Si LQ, Li PF, Yun ZH. A Method for Detecting the Quality of Cotton Seeds Based on An Improved Resnet50 Model. PLOS ONE. 2023 Feb;18(2):e0273057.

[4] Diaz-Escobar J, Ord´ o˜ nez-Guill´ en NE, Villarreal-Reyes S, Galaviz-Mosqueda A, Kober V, Rivera-Rodriguez R, Rizk JEL. Deep-Learning Based Detection of Covid-19 Using Lung Ultrasound Imagery. PLOS ONE. 2021;16(8):e0255886.

[5] Haitao C, Yu L, Yun Y. Research on Voiceprint Recognition System Based on ECAPA-TDNN-GRU Architecture. 2023 IEEE 2nd International Conference on Electrical Engineering, Big Data and Algorithms (EEBDA); 2023.

[6] Xin A, Haitao Z, Shuai Z. ASC Model Based on Feature Stratification and Multichannel ECAPA-TDNN. 2022 International Symposium on Advances in Informatics, Electronics and Education (ISAIEE); 2022.

[7] Kusumawati D, Ilham A A, Achmad A. Vgg-16 and Vgg-19 Architecture Models in Lie Detection Using Image Processing. 2022 6th International Conference on Information Technology, Information Systems and Electrical Engineering (ICITISEE); 2022.

[8] Shi ML, Zheng DL, Wu TH, Zhang WJ, Fu RJ, Huang, KL. Small Object Detection Algorithm Incorporating Swin Transformer for Tea Buds. PLOS ONE. 2024;19(3):e0299902.

(10) Comment: Some spelling and grammatical mistakes are found in the article that must be corrected. For example see spelling of “classifiy”, “labeled”, “analysises”, etc., and a grammatical mistake like “…achieving an low Equal Error”, “…combined in pair”, “…are formed. And then, 101 these sub-groups are”

Author Response:

Thank you for highlighting the spelling and grammatical errors in the manuscript. We acknowledge the mistakes you pointed out.

We have thoroughly revised the manuscript to correct these errors in the revised version.

Overall Changes Made:

Based on the reviewers' feedback, we have made the following major revisions:

1. Abstract

(a) we have corrected the "Semantic feature combination/parsing issue is one of the key problems in sound event classification." into"Semantic feature combination/parsing issue is one of the key problems in sound event classification for acoustic scene analysis, environmental sound monitoring, and urban soundscape analysis."

(b) Highlight all the modifications made.

2. INTRODUCTION

(a) We have added the rest of the article's contents.

(b) We have supplemented corresponding descriptions of the research objectives, aims, motivations, and ultimate beneficiaries.

(c) Highlight all the modifications made.

3. CONCLUSION

(a) We have supplemented "In future work, the voice recognizer confirmation system HCM-TDNN will be deployed in edge computing devices for real-time Urban environmental sound recognition scenarios."

(b) All modified sections are highlighted.

4.Others

(a) Enhance image clarity: We have improved the quality of full-text images by increasing the image resolution, using vector graphics, redrawing images and other methods.

(b) Improve figures caption: We have revised the manuscript to include more detailed descriptions after figures 4 to 7.

(c) Update headings: We have revised two repeated headings

(d) Add more cited references: We have supplemented more relevant models and expanded the list of cited articles.

(e) Polish the content of the article: The proofreading of the article has involved spelling and grammatical mistakes.

(f) Article formatting: The formatting of the article has involved modifications, including text alignment and paragraph formatting.

Conclusion:

We have taken into account all the feedback from the reviewers and are confident that our revised manuscript this time improves significantly upon the revised version. We hope the reviewers and editor will find our revisions suitable and consider our manuscript for publication in PLOS ONE.

Thank you once again for the constructive feedback and consideration. We look forward to your positive response.

Warm regards,

Jiwen Liang

Shenyang University of Technology
