# Supplementary material for: Hierarchical-Concatenate Fusion TDNN for sound event classification
Source: PLoS One. 2024 Oct 31;19(10):e0312998. doi: 10.1371/journal.pone.0312998 (PMC11527289; doi:10.1371/journal.pone.0312998)
Supplement: Attachment — Submitted filename: Response to Reviewers.pdf [file pone.0312998.s005.pdf]

Manuscript ID: PONE-D-23-39866R2

Title: Hierarchical-Concatenate Fusion TDNN for Sound Event Classification

Author name: Jiwen Liang, Baishan Zhao

Reply to the comments

Dear Martin Sagayam,

We thank the editor and reviewers for taking the time to review our manuscript and providing valuable feedback. We appreciate your careful consideration and constructive comments, which have helped us improve the quality of our work. Below, we address each of your comments and outline the revisions we have made. All the revisions have been marked in blue and red for a point-by-point response to the editor and reviewers' comments and highlighted in the manuscript with red background. All page numbers refer to the revised manuscript and below are our responses to the specific comments raised:

Comments from Reviewer：

Reviewer #1:

Comment: The author has addressed all the relevant queries. He has clarified the details with proper figures and tables. I recommend that you accept the paper for publication.

Author Response:

Thank you for your positive feedback on our manuscript. We appreciate your time and effort in reviewing our work.

Reviewer #2:

Comment 1: The abstract is poorly written. Most of the content focuses on the outcome of the proposed model. It should include a little bit of problem background and research objectives as well.

Comment: The objectives in the abstract are still missing.

Author Response:

Thank you for your constructive feedback regarding the abstract.

We acknowledge the shortcomings of the abstract, and we have re-written this part according to the suggestion. Specific modifications are as follows:

First, the objectives of the abstract have supplemented. We have added the "*The input audio signal in the acoustic scene classification is composed of multiple acoustic events, which usually leads to low recognition rate in complex environments. To address this issue,* this paper proposes the Hierarchical-Concatenate Fusion(HCF)-TDNN model by adding HCF Module to ECAPA-TDNN model for sound event classification."(please see page 1).

Comment 2: The literature review should be presented comparatively to show the research gap quantitatively (if possible). For instance, the author can identify some parameters and compare the related models/methodologies based on them in a tabular form.

Comment: The author is unable to present the comparative analysis in a tabular form based on certain parameters. Either it should be added or explained that this type of study cannot be represented in the suggested way.

Author Response:

We thank the reviewer's valuable suggestion regarding the Introduction section. We acknowledge the importance of a comparative literature review to effectively demonstrate the research gap and appreciate your recommendation to present this information quantitatively.

We have conducted a more detailed analysis of the existing models/methodologies and have identified key parameters for comparison. We have included a new table (Table 1) on page 3, which summarizes the performance of various related models based on these parameters.

Table 1. Comparative Analysis of Sound Event Classification Models.

Datasets	Method	Accuracy	Precision	F1-scores
TAU Urban Acoustic Scenes 2019	Conv-StandardPOST	77.2%	78.16%	77.7%
ESC-10	Two-Stream CNN	87.25%	85.1%	89.32%
Urbansound8k	Dual-Branch Residual Network	82.6%	80.7%	84.62%
Urbansound8k	Net50_SE	93.2%	96.1%	94.23%
Urbansound8k	ECAPA-TDNN	89.0%	84.0%	96.0%
ESC-50	CAR-Transformer	85.72%	95.21%	86.67%
CIFAR10-AV	SMMT	96.85%	99.48%	97.33%

Comment 3: The research objectives, aims, motivations, and ultimate beneficiaries should be well explained to show the significance of the research contribution.

Comment: It is still missing. The author just explained some of the aspects of the proposed methodology.

Author Response:

We thank the reviewer's helpful comments regarding our choice of using Mel spectrograms as input features in the proposed network.

Thank you for your insightful comments regarding the clarity of our research objectives, aims, motivations, and ultimate beneficiaries. We have carefully reviewed your feedback and have made the following revisions to address these points:

First, we have added the research objectives, motivations, and aims in the introduce section. Specifically, we have supplemented the research objectives "*The research objective is to optimize the ECAPA-TDNN network model architecture to achieve higher accuracy and reliability in different acoustic events.*"

We have added the motivations" *The complexity and diversity of acoustic events pose challenges for sound classification models.*"

we have added the aims" *The GoogleNet[48] is known to have a Multiscale-multichannel feature extraction architecture compared to other pre-trained models such as ResNet. In order to overcome the limitations of sound classification models in dealing with the complexity and diversity of acoustic events, we propose a novel HCF module based on the GoogleNet for the ECAPA-TDNN network model. Although the proposed model utilizes two well-established concepts (convolution and pooling), the*

combination of these two concepts for Multiscale-multichannel feature extraction architecture is the first work in sound event classifications. The combination of the convolution and HCF modules is expected to work complementarily to achieve better performance in SEC. The convolution module captures the convoluted spectrum features, whereas the HCF module captures the receptive field of spectrum. To evaluate the efficacy of our method, we have conducted experiments on UrbanSound8K dataset. The results show that our method imparts the stable performance."

Besides, we have clarified the ultimate beneficiaries in the conclusion section. Specifically, we have added the "*On the prospects of systems design, the optimizations targeted at embedded deployment can help realize real-time urban environmental sound recognition scenarios and edge computing solutions. This research makes notable progress in sound event detection, and the presented approach, analyses, and directions can be laying the groundwork to enable smart city design.*"

We deeply appreciate your meticulous attention to detail and strong communication skills, which have undoubtedly strengthened the overall quality of our manuscript.

Comment 4: It is suggested to add a little bit more description after each figure instead of giving their labels only. For reference, please see figures from numbers 4 to 7.

Comment: Need to be done. The description includes the detail of the figure e.g. its meaning, significance, and relevance to the ongoing discussion.

Author Response:

Thank you for your valuable suggestion regarding the description of figures. We have carefully reviewed your feedback and have made the following revisions to enhance the clarity and detail of the figure descriptions:

1. Fig 1: We have expanded the description of Fig 1 from "*A model architecture with the sound event classification model proposed by HCF-TDNN.*" to "*A model architecture with the sound event classification model proposed by HCF-TDNN. The HCF-TDNN branch consists of two convolution layers and three SE-HCF blocks stacked together with the Batch Normalization laver, followed by the attentive statistics pooling layer. Linear Unit (PReLU) serves as an activation function.*" Details can be found in Fig 1, page 4.

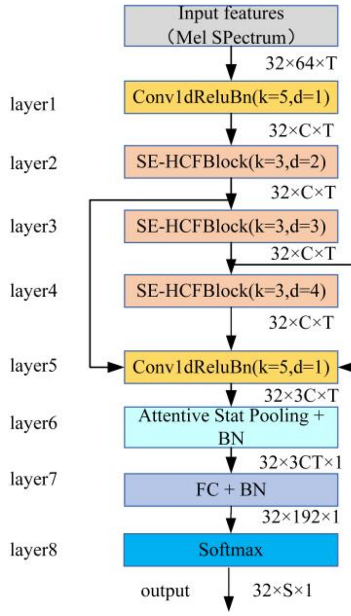

Fig 1. A model architecture with the sound event classification model proposed by HCF-TDNN. The HCF-TDNN branch consists of two convolution layers and three SE-HCF blocks stacked together with the Batch Normalization layer, followed by the attentive statistics pooling layer. Linear Unit (PReLU) serves as an activation function.

2. Fig 2: We have added "*Each HCF block consists of the stack of the layers that include Conv-BN-operations and the skip-connection. The processing step contains one-dimensional convolution of the pre-trained sound features over 3-second snippets for detailed feature recognition and capturing large targets.*" to the description of Fig 2. This has been revised on page 5.

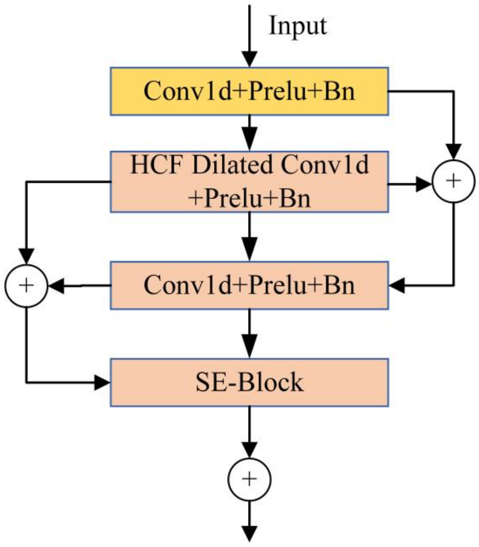

Fig 2. The SE-HCF Block of the ECAPA-TDNN architecture. Each HCF block consists of the stack of the layers that include Conv-BN-operations and the skip-connection. The processing step contains one-dimensional convolution of the pre-trained sound features over 3-second snippets for detailed feature recognition and capturing large targets.

3. Fig 3: We have included a more detailed description of the methodology and approach used in Fig 3. This has been revised on page 6. Specifically:

We have supplemented the "*The HCF module consists of several convolutional layers. Each layer contains a unique set of filters for extracting temporal features from the input audio signals. These filters are crafted to respond to different frequency ranges and time scales, allowing the module to capture a wide range of temporal characteristics in sound events.*"

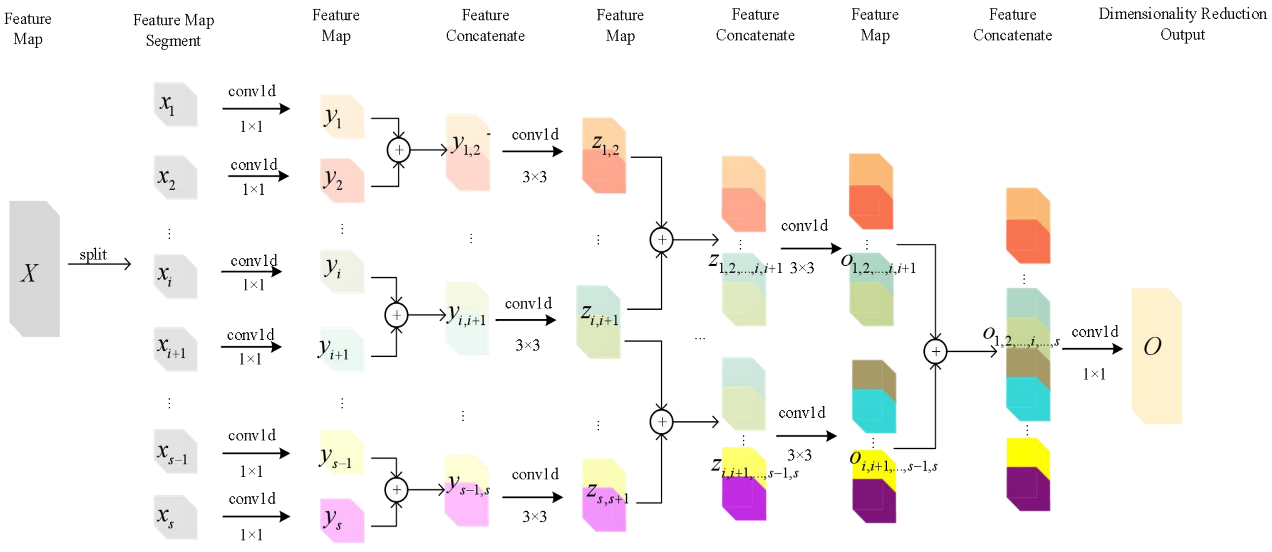

Fig 3. A Detailed View of the HCM. The HCF module consists of several convolutional layers. Each layer contains a unique set of filters for extracting temporal features from the input audio signals. These filters are crafted to respond to different frequency ranges and time scales, allowing the module to capture a wide range of temporal characteristics present in sound events.

4. Fig 4: We have enhanced the description of Fig 4. Details can be found in Fig 4, page 10. We have added " *The performance of sound category classification is meticulously detailed portrayals by each matrix. The matrices are clearly organized for easy comparison of TP, FP, TN, and FN across models. The strengths and weaknesses of models in sound type differentiation are indicated by the color gradients in each matrix.* "

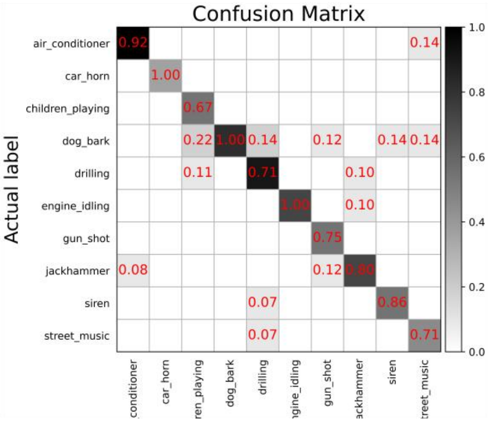

(a) Conformer

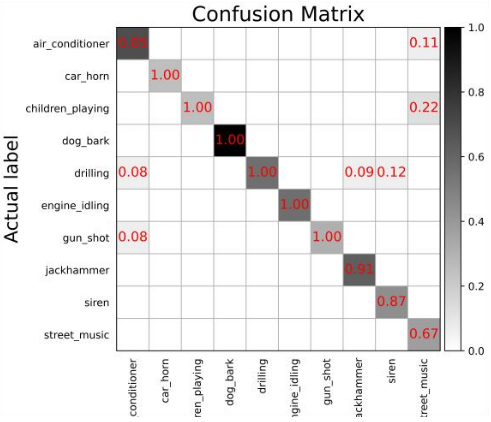

(b) ECAPA-TDNN

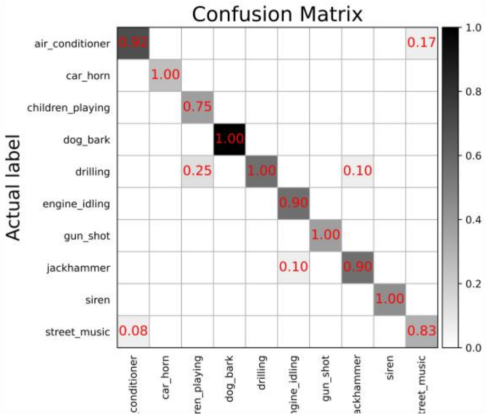

(c) AF-TDNN

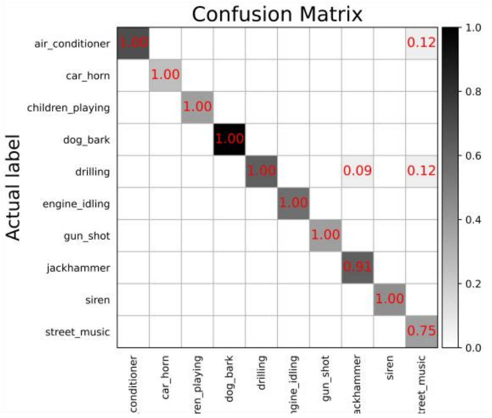

(d) HCM-TDNN

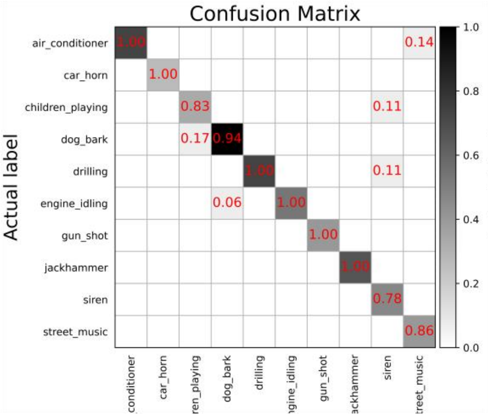

(e) ECAPA-TDNN-GRU

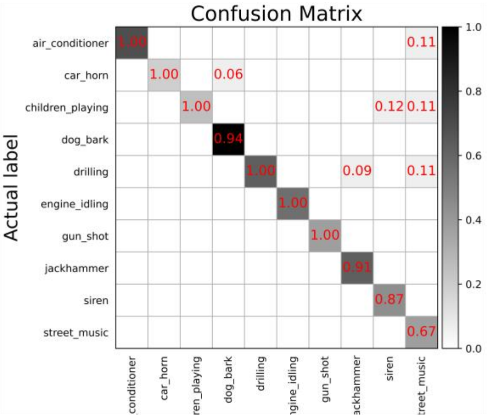

(f) Multichannel-ECAPA-TDNN

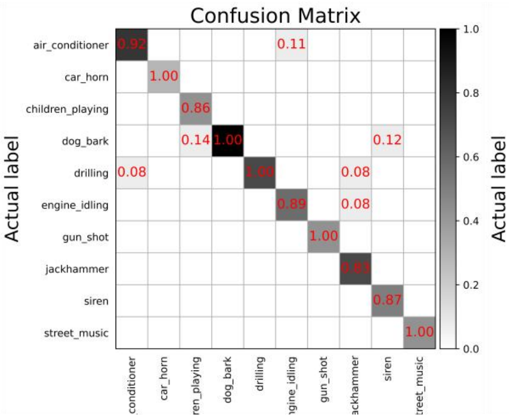

(g) VGG19

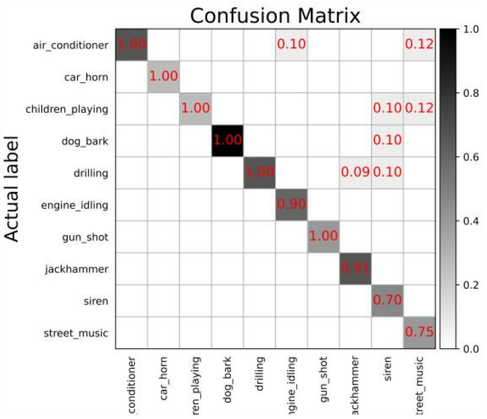

(h) STF-YoLo8

Fig 4. Comparison of confusion matrices for eight models in sound classification. The performance of sound category classification is meticulously detailed portrayals by each matrix. The matrices are clearly organized for easy comparison of TP, FP, TN, and FN across models. The strengths and weaknesses of models in sound type differentiation are indicated by the color gradients in each matrix.

5. Fig 5: We have expanded the description of Fig 5 from "*Box plot analysis of F1 scores for various models.*" to "*Box plot analysis of F1 scores for various models. In SEC, the F1 score range and potential outliers obtained by various models are vividly outlined by the block diagram for each model. The effectiveness of different sound scapes in complex tasks is distinguished by the visualization of box plots.*" Details can be found in Fig 5, page 11.

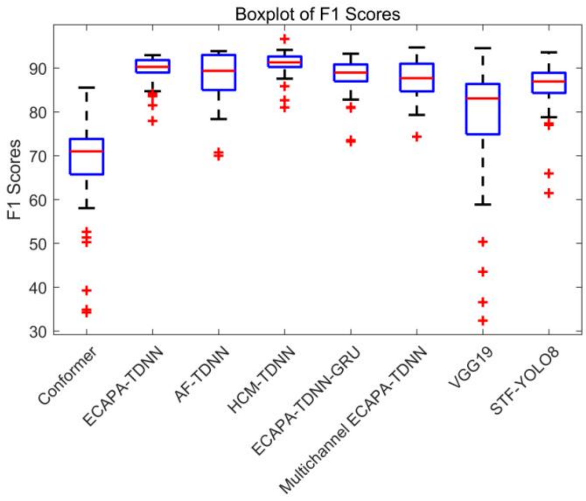

Fig 5. Box plot analysis of F1 scores for various models. In SEC, the F1 score range and potential outliers obtained by various models are vividly outlined by the block diagram for each model. The effectiveness of different sound scapes in complex tasks is distinguished by the visualization of box plots.

6. Fig 6: We have added " *The Kappa coefficients of multiple models within the sound classification domain are evaluated through the presented box plot. The distribution and consistency of Kappa scores are summarized graphically. The degree of agreement between actual and predicted sound classifications is measured by the box plot. The identification of models achieving higher precision and consistency in differentiating sound types is facilitated by the Kappa scores.*" to the description of Fig 6. This has been revised on page 11.

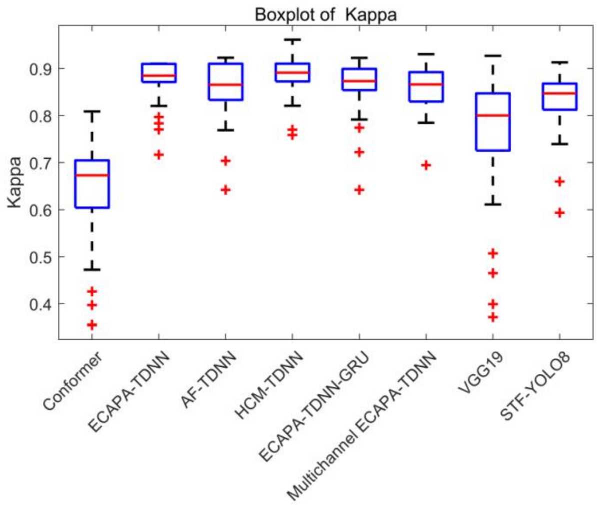

Fig 6. Comparison box plot of Kappa values for different models in sound classification. The Kappa coefficients of multiple models within the sound classification domain are evaluated through the presented box plot. The distribution and consistency of Kappa scores are summarized graphically. The degree of agreement between actual and predicted sound classifications is measured by the box plot. The identification of models achieving higher precision and consistency in differentiating sound types is facilitated by the Kappa scores.

7. Fig 7: We have included a more detailed description of the methodology and approach used in Fig 7. This has been revised on page 12. Specifically:

We have supplemented the "*The evolutions of each model's performance over different epochs are illustrated by the graph. The learning dynamics and convergence rates of various models are revealed by this visualization. A visual assessment of model convergence and training effectiveness is offered by the distinct learning curves. The models that struggle to minimize the loss are also identified by these curves.*"

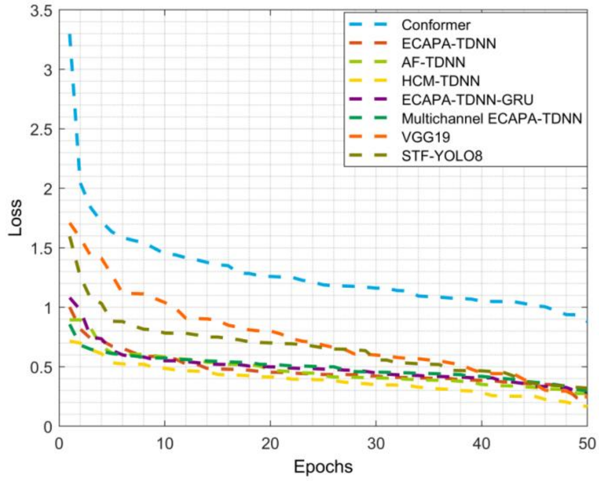

Fig 7. Comparison of Loss curves across various models on UrbanSound8K dataset. The evolutions of each model's performance over different epochs are illustrated by the graph. The learning dynamics

and convergence rates of various models are revealed by this visualization. A visual assessment of model convergence and training effectiveness is offered by the distinct learning curves. The models that struggle to minimize the loss are also identified by these curves.

8. Fig 8: We have enhanced the description of Fig 8. Details can be found in Fig 8, page 12. We have added "*Evolutions of the loss function over the epochs during the training using the training and the validation datasets for the different cases are evaluated. The testing accuracy curves also reveal the model's performance on SEC.* "

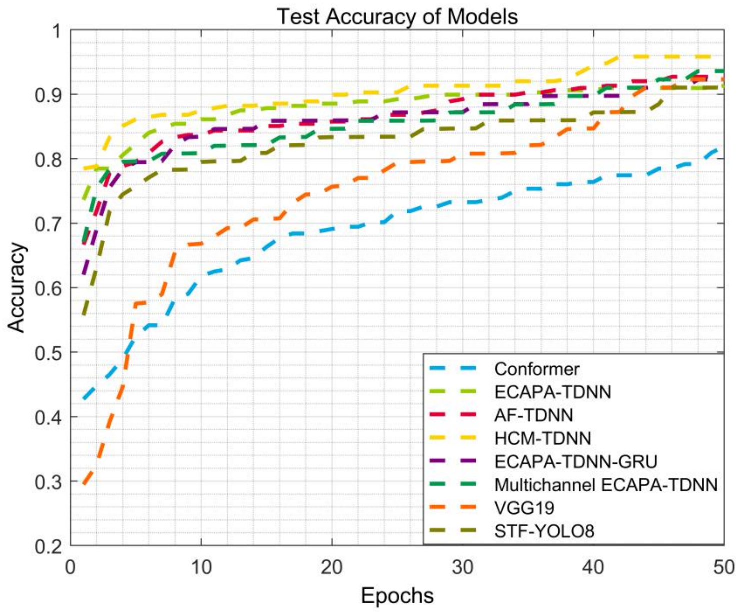

Fig 8. Model testing accuracy curves based on UrbanSound8K dataset. Evolutions of the loss function over the epochs during the training using the training and the validation datasets for the different cases are evaluated. The testing accuracy curves also reveal the model's performance on SEC.

Comment 5: Future directions should also be discussed to explain the possible future advancements in the underlined research area.

Comment: Future research directions are required instead of deployment details.

Author Response:

Thank you for your insightful comments regarding the inclusion of future directions in our manuscript. We have carefully reviewed your feedback and have made the following revisions to address this point:

Future Directions: We have added a new description to the conclusion of our manuscript in future work and beneficiaries. The conclusion has been revised on page 13. Specifically:

We have corrected the "*In future work, the voice recognition system HCF-TDNN*

model will be deployed in edge computing devices for real-time urban environmental sound recognition scenarios." in "*Future work should focus on further compressing the model design as well as incorporating additional shape and context information and exploring supplementary data sources.* "

We have also added ultimate beneficiaries for the future. Specifically: "*On the prospects of systems design, the optimizations targeted at embedded deployment can help realize real-time urban environmental sound recognition scenarios and edge computing solutions. This research makes notable progress in sound event detection, and the presented approach, analyses, and directions can be laying the groundwork to enable smart city design.*"

Comment 6: The referenced articles are fewer in number to better support the importance and significance of the underlying study. It is recommended to increase the number of referenced articles by adding more related models/methodologies to be compared with the proposed one.

Comment: It is hard to mention a reasonable number of references. But seems that there is still room to increase the scope of the literature review.

Author Response:

Thank you for your insightful comments regarding the number and variety of referenced articles in our manuscript. We have carefully reviewed your feedback and have made the following revisions to address this point:

References: We have expanded the list of referenced articles by adding more related models and methodologies to be compared with the proposed one. This has been revised on pages 2-3, where we now include a more comprehensive range of relevant literature that supports the importance and significance of our underlying study. Specifically:

We have added some relevant references of sound classification models.

1. Besides, the ResNet[16,17] and its variants[17-18] are combined with the SE module [18-20] to enhance the robustness of sound classification model.

2. With the rise of Convolutional Neural Networks (CNN)-based methods in various sound signal, such as TS-CNN10[19], ResNeXt_GAP[20], and AST-P[21].

Besides, we have added a discussion of more sound classification models.

1. For instance, Su et al. [22] proposed a TSCNN-DS model composed of two

four-layer CNNs to extract features from the LMC feature set and MC feature set, followed by classification. In a similar study, Sharma et al. [23] proposed an ADCNN-5 model composed of CNN and attention blocks based on DCNN and used a four-channel feature atlas composed of MFCC, GFCC. Some methods are based on shallow CNN architectures [24, 25] and have obtained some results in SEC tasks. However, shallow CNN architectures are limited by their network depth in extracting local high dimensional audio features, and cannot focus on features in the time-frequency domain over longer distances.

2. Motivated by the limitations of capturing long-range dependencies, several professionals have recently adopted the use of attention mechanisms to address the sound classification problem. Subsequent models like SMMT model[37], have combined attention mechanisms with spiking neural network to emphasize certain partial features to further improve the performance. Although it enlarges the perceptual field, increases the depth and ability of feature extraction of the network, the number of parameters is much larger than that of shallow CNN. The network model is also too deep to cause degradation and other problems more easily.

3. In audio processing-related tasks, feature correlation over long distances cannot be ignored. To obtain more discriminative and powerful information, Li et al. [38] proposed CAR-Transformer Neural Network Model.

Overall Changes Made:

Based on the reviewers' feedback, we have made the following major revisions:

1. ABSTRCT

(a) We have added research objectives. We have added the "The input audio signal in the acoustic scene classification is composed of multiple acoustic events. A low recognition rate of complex environments is led to by the composition of multiple acoustic events in the input audio signal."

(b) Highlight all the modifications made.

2. TRODUCTION

(a) We have added some relevant references of sound classification models.

(b) We have added a discussion of more sound classification models.

(c) We have supplemented the research objectives, motivations, and aims.

(d) We have presented the comparative analysis in a tabular form based on certain parameters.

(e) Highlight all the modifications made.

3. CONCLUSION

(a) We have added a new description to the conclusion of our manuscript in future work and beneficiaries.

(b) Highlight the modified parts

4.Others

(a) We have added a little bit more detail description of all figures.

(b) Highlight the modified parts

Conclusion:

We have carefully addressed all the concerns raised by the reviewers and believe that our revised manuscript substantially improves upon the initial submission. We hope that the reviewers and editor find our revisions satisfactory and consider our manuscript for publication in Plos one.

Thank you once again for the constructive feedback and consideration. We look forward to your positive response.

Warm regards,

Jiwen Liang
Shenyang University of Technology
