# Supplementary material for: Hierarchical-Concatenate Fusion TDNN for sound event classification
Source: PLoS One. 2024 Oct 31;19(10):e0312998. doi: 10.1371/journal.pone.0312998 (PMC11527289; doi:10.1371/journal.pone.0312998)
Supplement: Attachment — Submitted filename: Review Comments.docx [file pone.0312998.s002.docx]

Review Comments

(1) The abstract is poorly written. Most of the content focuses on the outcome of the proposed model. It should include a little bit of problem background and research objectives as well.

(2) The Introduction section should provide a brief introduction for the rest of the article's contents instead of the literature review-related contents.

(3) The literature review should be presented comparatively to show the research gap quantitatively (if possible). For instance, the author can identify some parameters and compare the related models/methodologies based on them in a tabular form.

(4) The research objectives, aims, motivations, and ultimate beneficiaries should be well explained to show the significance of the research contribution.

(5) The graphics quality of figures should be improved so that the textual information should be better readable.

(6) It is suggested to add a little bit more description after each figure instead of giving their labels only. For reference, please see figures from numbers 4 to 7.

(7) The two headings are being repeated. See line number 50 and 181.

(8) Future directions should also be discussed to explain the possible future advancements in the underlined research area.

(9) The referenced articles are fewer in number to better support the importance and significance of the underlying study. It is recommended to increase the number of referenced articles by adding more related models/methodologies to be compared with the proposed one.

10) Some spelling and grammatical mistakes are found in the article that must be corrected. For example see spelling of “classifiy”, “labeled”, “analysises”, etc., and a grammatical mistake like “…achieving an low Equal Error”, “…combined in pair”, “…are formed. And then, 101 these sub-groups are”
