# Supplementary material for: Hierarchical-Concatenate Fusion TDNN for sound event classification
Source: PLoS One. 2024 Oct 31;19(10):e0312998. doi: 10.1371/journal.pone.0312998 (PMC11527289; doi:10.1371/journal.pone.0312998)
Supplement: Attachment — Submitted filename: Review Comments - 2.docx [file pone.0312998.s004.docx]

Review Comments

(1) The abstract is poorly written. Most of the content focuses on the outcome of the proposed model. It should include a little bit of problem background and research objectives as well.

Comment: The objectives in the abstract are still missing.

(2) The literature review should be presented comparatively to show the research gap quantitatively (if possible). For instance, the author can identify some parameters and compare the related models/methodologies based on them in a tabular form.

Comment: The author is unable to present the comparative analysis in a tabular form based on certain parameters. Either it should be added or explained that this type of study cannot be represented in the suggested way.

(3) The research objectives, aims, motivations, and ultimate beneficiaries should be well explained to show the significance of the research contribution.

Comment: It is still missing. The author just explained some of the aspects of the proposed methodology.

(4) It is suggested to add a little bit more description after each figure instead of giving their labels only. For reference, please see figures from numbers 4 to 7.

Comment: Need to be done. The description includes the detail of the figure e.g. its meaning, significance, and relevance to the ongoing discussion.

(5) Future directions should also be discussed to explain the possible future advancements in the underlined research area.

Comment: Future research directions are required instead of deployment details.

(6) The referenced articles are fewer in number to better support the importance and significance of the underlying study. It is recommended to increase the number of referenced articles by adding more related models/methodologies to be compared with the proposed one.

Comment: It is hard to mention a reasonable number of references. But seems that there is still room to increase the scope of the literature review.
